# How Are Service Dogs for Adults with Post Traumatic Stress Disorder Integrated with Rehabilitation in Denmark? A Case Study

**DOI:** 10.3390/ani7050033

**Published:** 2017-04-25

**Authors:** Chalotte Glintborg, Tia G. B. Hansen

**Affiliations:** Department of Communication and Psychology, Center for Developmental & Applied Psychological Science (CeDAPS), Aalborg University, Kroghstræde 3, Aalborg 9220, Denmark; tia@hum.aau.dk

**Keywords:** service dogs, PTSD, animal-assisted therapy, psychological rehabilitation

## Abstract

**Simple Summary:**

The use of service dogs for adults with mental illnesses has become generally accepted. With reference to a single case study of a client with Post Traumatic Stress (PTSD), this study illustrates some of the potential advantages, but also note an important concern that appears to have gone unnoticed. The provision of service animals/therapy animals for adults with mental illnesses must be sufficiently informed by relevant knowledge and integrated with concurrent rehabilitation efforts. When it is not, it may contradict existing evidence-based treatments or unintentionally worsen conditions such as anxiety. This study argues that integration is possible and greater coordination efforts should be made.

**Abstract:**

A severe mental illness like Post Traumatic Stress Disorder (PTSD) is known to have psychosocial consequences that can lead to a decreased quality of life. Research in Animal-Assisted Therapy (AAT) has revealed that the presence of a dog can have a positive effect on health, e.g., increase quality of life and lessen depression and anxiety. However, canine companionship is not a catch-all solution. Previous research has revealed methodological limitations that prohibit any clear conclusions, as well as a sparsity of critical reflection in anecdotal reports and case studies, which means that more research is needed to contextualize the findings. There has been an increasing interest in animal-assisted intervention in Denmark in recent years. Previously, authorities could only grant service dogs to adults with physical disabilities, but now this has been extended to adults with mental illnesses. Therefore, it has become important to explore how these service dogs are incorporated into rehabilitation practices in mental health, and how rehabilitation professionals react to the use of service dogs. This paper is a case study of a person who suffers from PTSD. This study examines how the person describes the significance of having a dog during her rehabilitation process, and how this is integrated with existing rehabilitation. The case study has been developed based on a semi-structured interview. A Thematic Content analysis was used to reveal dominant patterns and categories. This study revealed a lack of communication and collaboration between public administration (social service), service dog providers, health rehabilitation services, and providers of psychological treatment. It also revealed limited access for the dog to public services, limited success in incorporating the dog into goal-directed treatment and rehabilitation procedures, a strongly felt emotional support from the dog, and a perceived stigma by having the dog wearing a vest with he words “mentally ill” printed on it.

## 1. Introduction

Post Traumatic Stress Disorder (PTSD) is a psychiatric trauma- and stress-related disorder. Symptoms include intrusive re-experiencing (i.e., flashbacks), avoidance of trauma-related stimuli and thoughts, negative alterations in cognition and mood, and changes in arousal and reactivity (e.g., hypervigilance and angry outbursts) [1]. PTSD has previously been classified as an anxiety disorder and also has high rates of comorbidity with depression and substance abuse.

PTSD can result in considerable work-related and social disabilities [2]. It has been recognized as a difficult disorder to treat, with high rates of dropout and non-response (up to 50%) [3]. Dropout, low compliance, and reluctance to enter therapy in the first place present particular challenges for this population because of the avoidance of trauma-related stimuli and thoughts inherent in the syndrome. Evidence-based treatment for the condition is available (e.g., Exposure Therapy). However, it is not commonly used by clinicians due to its level of discomfort for clients [4]. Therefore, finding alternative therapies is often recommended [5,6]. Integrating support from animals is an option worth exploring.

The inclusion of animals in psychological treatment is not new. The notion of Animal-Assisted Interventions (AAI) is broadly defined as any intervention that includes an animal as part of the process [7]. However, when it includes a professional, goal-directed therapeutic intervention, it is referred to as Animal-Assisted Therapy (AAT). Less structured activities with animals are referred to as Animal-Assisted Activities (AAA), and the trained animals that are provided to assist with daily life activities are termed Service or Assistance Animals.

There is an essential difference between service dogs and therapy dogs. In AAT, the majority of programs involve therapy dogs who visit institutions and participate with their owners in specially designed interventions. By contrast, a service dog constantly lives with the client who is their owner. Therefore, the demands put on therapy dogs versus service dogs are different.

A number of rehabilitation programs, especially in the U.S., involve animals to some degree. One of the most commonly targeted populations for these services is individuals who have experienced trauma, e.g., PTSD [8]. In 1999, Altschuler [9] called for the integration of animal-assisted interventions into treatments for PTSD. In 2005, Lefkowitz, Paharia, Prout, Debiak, and Bleiberg [10] presented a new model, the Animal-Assisted Prolonged Exposure (AAPE), to fulfill Altschuler’s vision. This model suggests combining Animal-Assisted Therapy with existing treatments for PTSD. A fusion of AAT and exposure therapy could encourage hesitant clients to participate in, and complete, the treatment, since they would be less anxious in the presence of their companion animals. However, it still remains unclear whether empirical data supports this practice, as claims of its effectiveness rely primarily upon anecdotal evidence. 

The presence of an animal has been shown to have positive effects on health, mood, and quality of life by increasing levels of oxytocin and other important anti-stress agents in humans [11,12].

When it comes to re-experiencing such as flashbacks, the presence of an animal is identified as a compassionate reminder that danger is no longer present [12]. In addition, the presence of an animal has been found to elicit positive emotions, countering the emotional numbing that clients with PTSD often feel [13,14]. Moreover, a review by O’Haire, Guérin, and Kirkham [15] supported short-term, subjective benefits of AAI for trauma, including reduced depression, PTSD symptoms, and anxiety. However, effect sizes ranged from small to large, and intervention procedures and research designs varied greatly, reflecting the preliminary nature of research in this area.

Companion animals may also act as social facilitators that can connect people and reduce social isolation for individuals [16,17,18]. Individuals who suffer from PTSD often face challenges of hyperarousal. Therefore, being with an animal may be a salient feature to reduce anxious arousal [19]. A recent review by Krause-Parello, Sarni, and Padden found canine assistance for PTSD in veterans to be a promising modality [20]. However, the authors raise concerns about lack of protocols, cost, availability barriers, and animal welfare, and call for additional, rigorous research to advance its use as a treatment.

By contrast, other studies have revealed unintended negative consequences of the presence of dogs in therapeutic settings. Rothberg and Collins [21] discuss the impact of a service dog being present during psychodynamic group therapy. It is important not only to understand the stress that the service dog can be under during the tasks assigned to it, but also to understand how its presence can affect other people in a group (e.g., persons who are allergic to or afraid of dogs). Therefore, it is important not only to consider animal welfare, but also to consider the needs and rights of other group members in order to minimize potentially negative impacts of a dog’s presence.

The use of service dogs for adults with mental illnesses is a recent development in Denmark. In 2009, the first Danish service dog was granted to a person with motoric disabilities with reference to the same rules as guide dogs for the blind. In 2012, service dogs for adults with mental illnesses became officially equalled in the legislation [22]. This means that they are recognized as aids for daily life activities, and can be provided by the Municipality. Owning a service dog brings certain rights that owning a companion dog does not. Accordingly, service dogs are allowed in restaurants, on buses, in food shops, in public administration buildings, and so on. 

To the authors’ knowledge, only one Danish provider of service dogs is accredited as a member of Assistance Dogs International. This organization has chosen not to expand into the area of mental illness. Since 2012, webpages from at least four Danish providers have offered service dogs for people with mental illness, but at the time of writing only two were active. One of these providers recruits puppies and subsequently trains them and provides them to clients. When a person has been granted a service dog by the authorities, the organization will visit and evaluate whether the person is competent to provide care for a dog and can thus qualify as a recipient. If the person is approved, he or she will receive a service dog, which is trained and certified by the organization. The other provider recruits dogs from dog shelters, and they also offer training of clients’ existing companion dogs to become service dogs. Both organizations are private, foundation-driven, and are run primarily by dog trainers and dog behavior consultants.

Despite the growing use of service dogs in Denmark for adults with mental illnesses, it has not yet been investigated how these service dogs are integrated with existing rehabilitation practices in mental health (e.g., how are they used in rehabilitation, how rehabilitation professionals react to their use, where they are allowed in practice, etc.). This case study seeks to contribute to this limited body of knowledge by exploring how the provision of a service dog was matched with the daily life demands of a client with PTSD.

Notably, some of the concerns laid out in this article regarding the feasibility and practical use of service dogs for mentally disabled are generalizable concerns (i.e., the use of service dogs as safety behavior that might compromise the efficacy of exposure therapy for PTSD; the risk for dog welfare posed by volatile symptoms). However, there are also concerns that may be specific to the Danish context (including the logistics of funding, access to public space, and the overt identification of “mental health” as the reason for the service dog’s presence).

## 2. Methods

The participant was recruited through convenience sampling [22], specifically through a Facebook group for persons with mental illnesses who also have service dogs. The interview was conducted by the first author on 20 December 2016, took place in the participant’s own home, and lasted approximately one hour.

The interview followed an interview guide (see Appendix) including questions about the participant’s experiences of having a service dog, how the dog supports her, how the dog is used in existing rehabilitation, etc. The interview was completed in Danish, tape recorded, and later transcribed. Quotes in this article are translated into English.

The analysis is based on Thematic Content Analysis. Transcriptions were carefully read in a process of line-by-line reading and open coding in accordance with Miles, Huberman, and Saldana [23]. Some of the dominant patterns arising from the case study will be explored in depth. The participant is a 44 year old Danish female with PTSD. To protect anonymity and confidentiality, names and identifying details have been altered.

## 3. Analysis

This analysis focuses Helen and her dog Samson, a four year old Cavalier King Charles Spaniel. Helen is 44 years old and lives on her own in a town house in a large Danish city. She explains that she has been diagnosed with PTSD due to childhood traumas and receives various rehabilitation services: 6 hours of home support per week (rehabilitation professionals visiting her house and helping her with daily activities), psychotherapy once a week, and physiotherapy once a week. Helen is not able to work and is therefore on a social pension. Helen became a dog owner in 2013 and she describes it like this:

*The reason (*why I became a dog owner*) is that I love dogs and I looked after my brother’s dog for three weeks and realized how much it helped me. The company, and I was not afraid to sleep at night….Safety and having responsibility for something other than myself. And also the physical contact, which was very hard and almost non-existent in relation to other people, but I could have physical contact with the dog.*

In this way, Helen already had positive experiences of how a dog could support her before she had her own dog and started to train him as a service dog. Her brother’s dog made Helen feel safe and she was able to have physical contact with another individual. Therefore, she decided to have her own dog, Samson. She got Samson from a kennel.

In 2015, Helen read about a service dog provider on Facebook. She contacted them and introduced herself and Samson. Her initial idea was that she might assist them with something. However, they returned her email with an invitation to have her own dog trained as a service dog. Although Helen was undergoing rehabilitation at that time, the rehabilitation professionals were not involved in this process, but they were informed by Helen and supported the idea.

Helen was already consulting a dog behavior specialist at the time because of some issues with Samson (specifically, he was afraid to be alone). The service dog organization knew the consultant and agreed to an arrangement in which the consultant trained Samson as a service dog, in accordance with their requirements . Helen was informed about their policies and their final Public Access Test (PAT). The PAT is to ensure that the dog is stable, well-behaved, and unobtrusive to the public, that the owner has control over the dog, and that the owner and dog do not pose a public hazard. Helen provided the service dog organization with a psychological assessment from her psychotherapist, and she was approved for the training. Like Helen, the majority of clients whose service dog is obtained through this organization have PTSD.

### 3.1. Having the Service Dog: A Parallel Process with Limited Communication

During the interview with Helen, several themes emerge. One of the major themes is the lack of communication between the service dog providers and the rehabilitation system. The Municipality will only become involved if there is an application for funding a service dog as an aid, and in this case will only provide the funding for the training. In Helen’s case, the only funding required was provided by the service dog organization and therefore there was no apparent communication between them and the Municipality. The communication between Helen and the service dog organization was also sparse. Helen only had email correspondence with them and never met representatives from the organisation until later. However, she would have welcomed more contact with the organization and also thinks that it would be helpful if they could link people with service dogs together so that the clients can exchange experiences.

### 3.2. Public Access: No Access for Service Dogs in Public Administration Buildings

Despite the fact that service dogs for adults with mental illnesses are equivalent in status with service dogs for those with physical disabilities, there are still issues with access. When Samson started the training, he received a vest bearing the words ”Service dog for mentally ill”. This would be considered a violation of basic confidentiality and privacy in countries such as the U.S., but it is not in Denmark. The stigmatizing nature of the text will be addressed further later in this paper. Despite the words, the vest does not automatically guarantee Samson’s access to buildings:

Helen: A vest where it says service dog, right?

Interviewer: In that way you can see it and he already has this (vest)?

Helen: Yes.

Interviewer: Is he allowed everywhere now?

Helen: No, you need to make agreements with those places you want to go to and then he also has a yellow sign, where it says “under training” with capital letters. But you make an agreement before entering, unless the shops are dog friendly already.

(…)

Helen: The hardest place to get access to with dogs is the Municipality. The most difficult place is the public administration offices (social services), “P1”, public administrations headquarters and citizen service, which is now also at “P1”, and the library; I never go there…they… I know that Peter (a development consultant and project manager) sometimes can arrange access by some means, and it’s really…but he has to talk with the guards every time…and I think it’s kinda…that you don’t have a greater understanding of this.

It is quite a paradox if the hardest places to gain access to with service dogs are the public administration buildings, given that the public administration is responsible for granting service dogs to clients. However, the question of access seems to be person-sensitive. Helen mentions an employee, Peter, who helps her and can explain to the guards that it is okay for Samson to enter the building, but this access only happens when Peter is there. Peter is an advocate for service dogs for people with mental illnesses, and has been working for the recognition of service dogs for the mentally ill in line with other types of service dogs.

Such person-sensitivity is also mentioned when Helen describes how the rehabilitation professionals react to Samson as a service dog and how they use him in the rehabilitation.

### 3.3. Integration of Service Dogs with Existing Rehabilitation?

When asked what the rehabilitation providers’ reactions were to Samson as a service dog, she answers:

Helen: My home supporters (social workers) think it is a good idea, and that’s it. It is not that they say they do not want to be involved, but they just aren’t. They do not even ask if he is certified yet.

Interviewer: Ok.

Helen: Well, they ask “are you soon finished with…is he done with the training” but they don’t ask “is there anything we can do” or “do you get your training done” or ehm… “is there anything we could do”…so there is nothing.

Interviewer: So it seems like bit of a parallel process.

Helen: Yes.

(…)

Helen: *Well, I did with Maria* (talk about Samson as a service dog) *from “Bogestien”* (the supported house where Helen lived before)*, because she knew, she saw the difference from before I had Samson to when I got him, and she also knew much more about what he could do, and used him.*

In this account, Helen describes a difference in how her supporters react to Samson. The majority just see him as a pet dog and not as a contributor to her rehabilitation process.

Later in the interview, she gives specific examples of how he is not involved in the rehabilitation, i.e., in achieving rehabilitation goals. One of Helen’s rehabilitation goals is to be able to go to the grocery store and shop without professional support. However, there is some incongruence in how to achieve this goal:

Helen: (…) they are pressuring me to practice shopping for groceries on my own.

Interviewer: Yes.

Helen: But I don’t shop for groceries without my dog, so this is my choice, right.

Interviewer: Yes.

Helen: But, ehm, but but they haven’t…we haven’t made a plan at the moment. There is a lot of pressure, they think we must start the training to get this solved, but then I say, “we have to wait until Samson is done with his training, and he can come along, then we can slowly start the training”.

The professionals are goal oriented and push for things to happen. One interpretation could be that in a rehabilitation process, time is money for the Municipality. Accordingly, the professionals cannot wait for Samson to become ready to accompany Helen, and maybe they do not see him as an important factor in this achievement either. Another possibility is that they perceive Helen’s postponement of her training as avoidance, since this is a core symptom of PTSD. However, Helen strongly wants to combine the two things and is adamant in saying that she will not go to the store without Samson.

In the interview, Helen says that the professionals might think that it would be fine if she could go to the shop with Samson, because this would mean that Helen would be capable of doing it herself, in accordance with her rehabilitation goals. Increased autonomy is often a goal in rehabilitation practice, for reasons of client recovery and empowerment, and it could also be argued to be in the economic interest of the authorities to have more capable citizens in this respect. Nevertheless, the professional does not actively support the integration of Samson as part of this process, and Helen is unwilling to try without him. However, Helen would like the professionals to integrate themselves more into the process of Samson’s training as a service dog. Perhaps doing so would have accelerated this process. 

Helen says that her dog trainer has agreed to advise the professionals on how they can help the promotion of Samson as a service dog, but the professionals have not shown interest in this. The main problem may be that they do not see it as part of their job they support humans and not human-animal relations. Also, they may expect advice to be uninformed and professional confidentiality could make communication difficult.

Since Helen also sees a psychologist, she was asked how Samson is integrated into psychotherapy.

Interviewer: What about your psychologist?

Helen: The psychologist does not really get into it either, but she thinks it is ok that I bring him to therapy and he just lies quietly in his basket.

Interviewer: But does she use him actively in therapy?

Helen: Once in a while she does.

Interviewer: Ok, can you give some examples of how he is used?

Helen: Hmm, there has been times where we have talked about difficult things, and I have become a little quiet and distant, then she has said can you try and call for Samson.

Interviewer: So he has been used in therapy?

Helen: Yes, but not normally. If we just have an ordinary session, he will just be lying next to me in the basket or in his bag. If I become uncertain, I will put a hand down to him.

Interviewer: Yes.

Helen: And that’s ok, he is also allowed to walk around and sniff if he wants to, but he usually doesn’t.

It is okay for Samson to join the therapy session and also to walk around. He is used sometimes during the session as a tactile soother. However, the psychologist does not ask how Helen uses Samson at home, nor does the psychologist use him actively in the PTSD treatment.

### 3.4. The Support Provided by Samson

As previously mentioned, Helen had some experience with what a dog could do for her, which was the reason why she became a dog owner. There are various things that Samson assists her with, as described in the following excerpt:

Interviewer: Can you describe some of the thing that Samson can assist you with?

Helen: There are a lot of things…one of them is to help me navigate. (She laughs) He is great at finding his way, and that provides a general safety net…when I walk on the street without Samson, then I’m 10 times more insecure about what is going to happen. But when I walk with him, I focus on him and whether anybody steps on him, if he is with me and walks nicely besides me….So my focus is on him and it is also easier for me to be with people because it takes some of the focus from me. People would like to talk about him, and I would like to talk about him.

Interviewer: So it is easier to go out?

Helen: Yes, and I sleep better when he is there.

Interviewer: Yes, so he also helps you at home?

Helen: Yes, and so he is just…for instance, when I need to take a shower, I do not really like that either, but when Samson is there, I like to do it and everything is fine. Then he lies on the bathroom floor and walks away when I’m done (she laughs).

Interviewer: So his presence makes you safe?

Helen: Yes.

(…)

Helen: Yes, I don’t know how he…he also helps me with flashbacks.

Interviewer: What does he do?

Helen: He scratches the door, as if he wants to go out, because then he knows I will get up and react to things, or he jumps up.

Interviewer: Does it help you to get out of the flashbacks?

Helen: Yes.

In this citation, five supporting functions are revealed: (1) Samson is a *social catalyst*, (2) Samson allows *ventriloquizing*, (3) Samson makes her *safe*, (4) Samson is a *distractor* when she has flashbacks, and (5) Samson is a *navigator* for her so that she does not get lost.

Even though Samson is a small dog, Helen conceives of him as a dog that makes her safe. He could not effectively defend her because of his small size and gentle temperament, but nevertheless his presence alone makes Helen feel safer. In psychological terms, this could be described as Samson being used by Helen for safety behavior [24]: as a coping behavior to reduce anxiety and fear in uncomfortable or anxiety-provoking situations, such as going to the shopping centre or taking a shower. However, especially from a long-term perspective, safety behavior is potentially counterproductive in the treatment of PTSD, a point that will be returned to in the discussion.

Helen relies on Samson’s abilities to help her, and she mentions several occasions on which he has helped her get out of flashbacks, even when these happen on their walks. She gets very disoriented afterwards, but then he leads the way home. She trusts him in this and therefore feels safe to go out, despite the risk of sudden flashbacks.

Samson has not been trained to do these things, but she describes him as a sensitive dog who is closely connected to her. She thinks that he might sense things and instinctively react to them.

Samson also serve as a social catalyst and a tool for ventriloquism. It is easier for Helen to meet other people “through” Samson. She can talk about him and shift focus to him instead of herself. She can also purportedly speak on his behalf to communicate her own needs (ventriloquizing, cf. Tannen [25]). During the interview, Helen and the interviewer also talk about Samson and address him, since he is on a chair at the kitchen table. In this way, he is also part of this interview. She gives examples of how she talks through him (ventriloquizes) because it is easier for her than to express things directly to other people. At the end of the interview, we saw an example that illustrates this function, when she addressed Samson with the words, “*You’re tired now, Samson”.*

### 3.5. Labelling Vest ”Service Dog for Mentally Ill”

Near the end of the interview, Helen gets up and wants to show the interviewer the vest that Samson needs to wear as a service dog. The vest bears the text “Service dog for mentally ill”. Helen finds this severely stigmatizing, saying that, “*You do not want to flaunt it like that*”, (that you have a mental disorder) and that, “*It is like a sign for a psychiatric hospital*”. She would prefer the vest to just say “Service dog”. Several others who also have service dogs from the same organization agree, she says. They brought this concern to the organization, but were told that the organization considers it important that people know what kind of service dog a given individual is. However, Helen still finds it very disturbing, and so do others. She remarks, “*There are actually also some of my former home supporters that questioned it* (the text)”.

## 4. Discussion

This case study supports findings from previous research that the presence of an animal can reduce anxiety and elicit positive emotions [11,12,13,14]. When Samson accompanies Helen on walks or when she takes a shower, she feels *safe* and is able to do it. Accordingly, this can be identified as Samson supporting Helen’s safety behavior. Safety behaviors are overt or covert actions performed to prevent, escape, or minimize a feared catastrophe or associated distress [24].

In addition, it was also found that Samson serves as a *social catalyst* [11,25]: he facilitates Helen’s interpersonal interactions. There is a large body of research on how a pet dog can act as catalysts for human social interactions, and it has been suggested that this may enhance feelings of well-being and improve psychological health by the dog acting as a social lubricant, encouraging relationships with people, etc. [26]. In addition, it has been shown that, even in unfamiliar places, the companionship of a dog can enhance social contact between a dog owner and other people. This is in line with what was found in this single case study. When Samson is with Helen, she finds it easier to interact with other people. In a similar vein, she also uses him for *ventriloquizing*. Deborah Tannen [27] employs Bakhtin’s concept of *ventriloquizing* to describe the discursive strategy a person uses when he or she talks through a nonverbal participant (e.g., a dog), as exemplified by telling Samson that he is tired, which indirectly sends a message to the interviewer.

In this way, this study’s findings resonate with previous studies that demonstrated benefits of animal assistance. However, despite the support a dog can provide to clients with mental illnesses and the fact that service dogs can now be provided for this group of people, this analysis also reveals that these dogs are not integrated with the rehabilitation of clients suffering from PTSD. This fact raises two concerns discussed in the following subsections.

### 4.1. Contradictions in Psychological Rehabilitation

Even though Exposure Therapy is an efficient and recommended intervention for adults with PTSD, there have been some concerns about its use because of the levels of discomfort it causes for clients. At the same time, safety behaviors have been described as a major cause of continuing anxiety and a reason why people may not desensitize to unrealistically feared stimuli during exposures [28]. Multiple studies have shown that safety behaviors can harm people‘s abilities to get past anxiety in certain situations [28,29,30,31]. In other words, anxiety might be maintained or even worsened if a person continues to perform safety behaviors, because he or she is unwittingly reinforcing the idea that the situation is very dangerous. Thus, safety behavior can be supportive in the short term, but becomes maladaptive in the long run since it can sustain or escalate anxiety and fear of nonthreatening situations.

The treatment model suggested by Lefkowitz, Paharia, Prout, Debiak, and Bleiberg [10] called Animal-Assisted Prolonged Exposure (AAPE) proposes a combination of animal-assisted therapy with exposure therapy. The rationale for the model was that clients would be more willing to put themselves into a feared situation if they were accompanied by a dog. A more recent study by Hunt and Chizkov [32] provides some analogous evidence for Lefkowitz et al.’s proposal of adjunct AAT to prolonged exposure. Moreover, they found that the dog’s presence seemed particularly helpful to more introverted individuals, enabling them to reap the same long-term rewards as their more extraverted peers in terms of decreasing depressive symptoms.

This was also seen in this case study. Helen will go on walks and take showers when she is accompanied by Samson. In this way, she exposes herself to the fear of these things. The feelings of safety induced by a dog can help adults with PTSD challenge their fears and can lead to a new evaluation of the world as relatively safe and of themselves as able and strong [10]. However, an important caveat is the danger of the person misattributing causality to the dog. A recent review by Blakey and Abramowitz [33] (p. 13) stated that, “*although safety behaviors are not unconditionally deleterious, they tend to interfere with exposure outcomes, possibly by promoting safety misattributions, preventing therapeutic information processing, or interfering with other mechanisms central to inhibitory learning theory*”.

To avoid this misattribution, it is important that the use of animal assistance to deal with PTSD related fear is done in close cooperation with a therapist. Accordingly, the next step in therapy is, as happens with human supporters, that the animal assistance should be faded out progressively with each exposure session.

Since there was no integration with ongoing rehabilitation, this has not happened for Helen. Helen continues to use Samson in these situations, which might lead to contradiction in treatment and in the worst case maintaining or worsening her anxiety. In the interview, Helen reveals that she is aware that she is receiving two types of treatment, in that she designates the interactions she has with Samson as treatment, and she indicates a potential conflict with the agreement she has with the psychologist, as she acknowledges that, ”*when you enter psychotherapy you agree that you are not receiving any other treatment at the moment*”.

In a similar vein, a disconnection is seen between the two “treatments” when it comes to dealing with flashbacks. Helen’s flashbacks are not currently addressed in her psychotherapy. Depending on the therapeutic paradigm, this could be a deliberate choice or simply not a high priority at the moment. However, as illustrated in the interview, Samson helps her get out of flashbacks in her everyday life. Dogs can be used in dealing with flashbacks in therapy too. Frightening memories returning in the form of flashbacks represent another core symptom of PTSD, and these flashbacks may persist if the only approach taken to coping with them is trying to push them away (avoidance provide short-term relief at the cost of long-term persistence). The presence of a dog in a therapy session dealing with trauma cannot eliminate anxiety, but the dog can help the person to engage in the process and facilitate habituation [10]. This was also seen in a study by Reichert [34] dealing with adult abuse survivors and in a study by Dietz, Davis, and Pennings [35] dealing with child sexual abuse. The first step to voluntarily recalling the memories was to whisper the details of the assault in the dog’s ear. During this, clients pet the dog as a soothing, tactile, and grounding comfort. Clients are slowly confronted with painful memories, a process that includes cognitive engagement, i.e., recalling details and emotional engagement with the associated fear and anxiety. The clients allow themselves to feel and to experience the memory while in control and thus systematically habituate themselves to the fear and anxiety [34,36]. The act of petting Samson was also used in Helen’s therapy sessions, not to recall memories, but purely as a source of comfort, and apparently not systematically.

In summary, there are numerous possibilities in animal-assisted rehabilitation. However, the process needs to be combined with psychological rehabilitation in order to diminish the risk of misattribution, and of unwittingly reinforcing the symptoms one seeks to extinguish, etc. Therefore, the use of service dogs for adults with PTSD (or any other mental illness) needs to happen in close cooperation with rehabilitation professionals. Ideally, rehabilitation professionals should be educated about the opportunities presented by animals in rehabilitation, both in the form of AAT programs and service dog programs. Furthermore, all providers involved need to cooperate closely so that the dog becomes part of a professionally informed treatment plan rather than evoking parallel treatment processes that in the worst case may contradict each other. In this connection, it is noteworthy that anyone may provide service animals, as there is no national inspection, supervision, or accreditation for providers. This leads to a second concern: is current knowledge and awareness sufficient throughout the system?

### 4.2. The Need for Expertise and Standards

Service dogs are currently defined and granted as a helping aid under Danish legislation. However, when the use of service dogs is extended to a new population (adults with mental illnesses), there is a need to re-evaluate whether they are still just helping aids to support daily activities in the same way as for the physically disabled. This case study illustrates that they become more complex agents than just a helping aid when their task is mental support. This implies a need for increased knowledge and awareness. However, this study raises some concerns about the level of knowledge as well as the lack of cohesion between service dog associations and existing rehabilitation practice in general, and also how the use of service dogs is supported in Denmark. In addition, it raises a particular concern about the provision and use of service animals/therapy animals for adults with mental illness when it is not sufficiently informed by relevant knowledge and integrated with existing rehabilitation, since it can contradict existing treatment or unintendedly worsen a current mental state like anxiety.

In Animal-Assisted Therapy, evidence-based programs exist that might be applicable. The case study focused on Helen’s service dog, but a psychotherapist might also find an animal-assisted psychotherapy program useful, e.g., Animal-Assisted Prolonged Exposure. Professionals also need to be better informed of the potentials (and pitfalls) of including a therapy dog or service dog and how to coordinate their expertise with that of others for the development of relevant strategies and guidelines.

Internationally, standards and best practice guidelines have been developed over the last decade, e.g., by The International Association of Human-Animal Interaction Organizations [37], Assistance Dogs Europe [38] and Animal-Assisted Intervention International [39], and it is relevant to require education, accreditation, or at least commitment to such standards as a prerequisite for funding animal-assisted therapy and service dogs. 

### 4.3. Awareness Needs

Tedeschi et al. and Krause-Parello, Sami, and Padden [20,40] have previously noted concerns that safety and animal welfare may not be adequately taken into consideration when the use of therapy and service animals expands into new fields, such as mental illnesses, including PTSD as in the present case study. There is also a lack of awareness of the need to integrate psychologically impactful interventions, including service dogs, with other interventions that are provided to support the same people by different means. In the case described above, it seems clear that neither the animal welfare needs nor consultation and integration with existing therapeutic treatment have been given sufficient thought. This study strongly recommends future efforts to address this oversight. 

In mental health treatment, much effort in recent years has been put into developing an evidence base for which treatments work and which are questionable [41,42] or even unintendedly harmful [43]. There is a similar need to develop an evidence base in Animal-Assisted Therapy [44,45] and in the use of service dogs to support rehabilitation. To achieve their potential benefit and avoid predictable pitfalls, the provision and use of dogs for mentally ill clients must be informed by this evidence base. In the case of PTSD, programs have been developed with this need for integration in mind [10], and the authors of this study hope to see similar programs developed for the use of service dogs in rehabilitation. For both areas, the inclusion of expertise on animal welfare is also required, as is supervision for service dog providers, service dog recipients, and handlers in Animal-Assisted Therapy.

Finally, another issue that has caused much concern in recent years is the fragmentation of services offered for mental health concerns in primary and volunteer-based sectors, the risk of these counteracting each other, and of important needs being overlooked in the false assumption that other entities dealt with them. The Convention on the Rights of Persons with Disabilities [46] strongly advises that rehabilitation efforts are coordinated in order to ensure comprehensive rehabilitation based on multidisciplinary efforts in which professionals each use their area of expertise. In the area of Animal-Assisted Therapy and the provision of service dogs for the mentally ill, the relevant areas of expertise include professional knowledge of mental illness, client support and empowerment, animal training and welfare, and how to coordinate all of these elements.

## 5. Conclusions

This case study illustrates and supports previous findings that a client with anxiety issues such as those arising from PTSD can achieve support and relief through the use of a service dog. However, it also reveals that service dogs may be provided with little consideration of the context into which they are sent. To increase benefits and reduce pitfalls of the provision of service dogs for people with mental illness, their use must be integrated with professional knowledge and treatment efforts in a coordinated rehabilitation plan.

## Appendix A

**Interview guide**

- Can you elaborate on why you became a service dog owner?
- Did you already have the dog?
  ○ If yes: why did you become a dog owner? Why this breed?
- What is your need for a service dog?
  ○ What kind of mental disabilities do you have?
- Where did you first hear of service dog providers?
- Where was your dog trained?
- Who initiated the service dog training?
- Who paid for the training?
- Did you receive support (rehabilitation service/psychological treatment, etc.) at that time?
  ○ If yes: were these professionals involved in the process of having a service dog?
- What happened after the certification?
- Will there be later certification/checkups too?
- How does your dog support you?
  ○ Can you describe things it does for you/with you?
  ○ Can you describe your daily activities with the dog?
  ○ What happens when you have a bad day?
- What has the reaction been from others?
  ○ Relatives and friends?
  ○ Rehabilitation professionals?
- How do you experience access to shops, public administration, etc. when accompanied by a dog?
  ○ Do you need to show documentation?
  ○ What are the reactions?
- Do you receive any other support?
  ○ How is your dog used in existing rehabilitation?
  ○ How is he used in your psychological treatment?
- Have you missed anything as a service dog owner?
